# Implication of Cytotoxic* Helicobacter pylori* Infection in Autoimmune Diabetes

**DOI:** 10.1155/2016/7347065

**Published:** 2015-12-28

**Authors:** Alessandro P. Delitala, Giovanni M. Pes, Hoda M. Malaty, Gavino Pisanu, Giuseppe Delitala, Maria P. Dore

**Affiliations:** ^1^Azienda Ospedaliero Universitaria di Sassari, Sassari, Italy; ^2^Department of Clinical and Experimental Medicine, University of Sassari, Viale San Pietro 8, 07100 Sassari, Italy; ^3^Baylor College of Medicine, Houston, TX, USA

## Abstract

*Background.* Type 1 diabetes (T1D) and type 2 diabetes (T2D) have been linked to* Helicobacter pylori* infection, although results are conflicting. No previous study addressed a possible link between* H. pylori* infection and latent autoimmune diabetes in adults (LADA). In this study, a correlation among* H. pylori* infection and the risk of autoimmune diabetes in comparison with T2D was investigated.* Methods.* Sera from 234 LADA patients, 105 patients with late-onset T1D, and 156 patients with T2D were analyzed for anti-*H. pylori* and the cytotoxin-associated antigen (CagA) IgG antibodies*. Results. H. pylori* seroprevalence was comparable in LADA (52%), late-onset T1D (45%), and T2D (49%) with no gender differences. The seroprevalence of CagA IgG was significantly higher in autoimmune diabetes (late-onset T1D: 45%, LADA: 40%) compared to T2D (25%; *p* < 0.028).* Conclusions.* Although* H. pylori* seroprevalence was similar in LADA, T1D, and T2D, anti-CagA positivity was significantly increased among patients with autoimmune diabetes, suggesting that more virulent* H. pylori* strains might be a trigger for immune mechanisms involved in their pathogenesis.

## 1. Introduction

*Helicobacter pylori* colonizes approximately 50% of the world's population. Differences in prevalence relate to age, socioeconomic status, and geographic location [1, 2].* H. pylori* infection is commonly associated with gastritis, gastric cancer, and peptic ulcer disease, as well as with a variety of extragastric manifestations [3–5]. The infection elicits a robust inflammatory response [6] that in turn may result in molecular mimicry, which may be responsible for some of the extragastric manifestations [4, 5]. Available data also suggests that* H. pylori* infection might be associated with diabetes mellitus.

The relationship between* H. pylori* infection and development of diabetes is thought to be possibly mediated by the long-standing chronic inflammation which has been implicated in insulin resistance [7, 8]. A recent prospective study demonstrated an association between* H. pylori* infection and the rate of incident diabetes [9]. The authors analyzed 782 Latinos over 60 years of age without diabetes living in California in 1998-1999. Sera were tested for antibodies against herpes simplex virus 1, varicella virus, cytomegalovirus,* H. pylori*, and* Toxoplasma gondii*. Subjects were followed up until June 2008 and the relative incidence rate of diabetes in relation to* H. pylori* IgG status was evaluated. Individuals positive for* H. pylori* infection at the enrollment time were 2.7 times more prone to develop diabetes than seronegative individuals [9].

There are several reports describing an association between* H. pylori* infection and autoimmune diseases [10]; however, evidence of a link with type 1 diabetes (T1D) is conflicting. For example, Pocecco et al. reported increased prevalence of* H. pylori* with age in young diabetics [11], while according to other studies the frequency of* H. pylori* infection in T1D was comparable to healthy controls [12–14]. Moreover, an increased frequency of* H. pylori* reinfection following treatment in comparison to nondiabetic dyspeptic patients was observed, suggesting differences in susceptibility [15].

Latent autoimmune diabetes in adults (LADA) is a type of autoimmune diabetes that resembles T2D at onset. LADA represents 5–10% of subjects previously diagnosed as having T2D with which it shares some phenotypical features [16]. LADA is characterized by a later onset and slower progression towards insulin dependence than typical T1D.

The role of* H. pylori* infection in T2D is unclear [6, 12, 17] and it is still debated whether* H. pylori* has a pathogenic role or whether diabetic patients have an increased susceptibility to* H. pylori* infection. No previous studies have examined the association between LADA and* H. pylori* infection. Therefore, we investigated the prevalence of* H. pylori* infection in patients with autoimmune diabetes (both LADA and late-onset T1D), as well as nonautoimmune T2D.

## 2. Materials and Methods

### 2.1. Study Population

Demographic features of LADA patients from Sardinia recruited in this study have been reported previously [18, 19]. Briefly, a total of 5,568 Sardinian patients with T2D at diagnosis were screened for the presence of pancreatic islet autoantibodies. These patients have been referred to as a part of a prospective longitudinal multicenter study, among the major diabetic units of the island (Sassari, Cagliari, Nuoro, Oristano). From the original cohort of 251 patients, 17 subjects were excluded because their sera were no longer available. A total of 234 serum samples, 126 women and 108 men (median age at onset of diabetes was 54 years, range 30–86 years), were analyzed. Diagnostic criteria for latent autoimmune diabetes patients were (i) presence of circulating glutamic acid decarboxylase 65 antibodies (GAD65Ab), (ii) age at onset of diabetes above 30 years, and (iii) absence of insulin treatment for at least 8 months after diagnosis. In addition, none of the patients presented with ketoacidosis and/or significant weight loss [18].

According to the study design, serum samples from 105 late-onset T1D patients (55 males, 50 females, age range from 39 to 55 years) were also analyzed. Diagnostic criteria for late-onset T1D were sudden onset above the age of 30 and presence of ketoacidosis [18]. Sera from 156 (85 males and 71 females, range 48–77 years) type 2 diabetic patients who resulted to be GAD negative at the antibody screening were randomly selected as controls for comparison with autoimmune diabetes.

The study was approved by the local ethics committee and all participants provided signed informed consent to participate in the study.

### 2.2. Serologic Methods

Blood venous samples were collected between 7 and 8 a.m., after an overnight fast. Serum samples were stored at −80°C until being assayed.* H. pylori* status was evaluated by an enzyme-linked immunosorbent assay (ELISA) for anti-*H. pylori* immunoglobulin G (*Helicobacter pylori* IgG, ELISA kit, Genesis Diagnostics Ltd., Littleport, UK), with a reported sensitivity and specificity of 99.2% and 90.9%, respectively [20]. In addition, the presence of putative* H. pylori* virulence factor was assessed by a specific serological ELISA test for IgG antibodies against CagA (CagA IgG ELISA Kit, Genesis Diagnostics Ltd., Littleport, UK), with a sensitivity of 96%, specificity of 97%, and interassay coefficient of variation of <12% [20].

### 2.3. Genotyping of Immune-Related Gene Variants

HLA class II and CTLA-4 genotypes, previously associated with the immune response in LADA patients [18, 21], were also analysed in relation to* H. pylori* infection. In LADA patients, HLA class II genotypes, determined by dot-blot analysis [18], were ranked as low, intermediate, or high risk for diabetes. At least two high-risk haplotypes were necessary to classify a patient in the high-risk category; the presence of one high-risk and one permissive-neutral haplotype was taken as the hallmark of the intermediate-risk category whereas carriers of two copies of negatively associated haplotypes or combinations of negatively associated and neutral haplotypes were included in the low-risk category. In addition, a G6230A (rs3087243) functional polymorphism within the CTLA-4 gene, exhibiting regulatory properties on immune effector T cells, was also genotyped in LADA patients. (Patients carrying at least one G allele were classified as high risk whereas patients with no G allele were considered at low risk.) Further, a total score for genetic risk was calculated combining both the HLA (low, intermediate, and high risk) and CTLA (low, high risk) categories, namely, by giving 0 to 5 points to the six progressively rising risk levels resulting from paired HLA/CTLA-4 assortment. Prevalence of CagA antibodies positivity was stratified according to this score.

### 2.4. Methods of Analysis

Subjects who were positive for anti-*H. pylori* and CagA IgG antibodies were classified into 3 groups: (1) patients with T2D, (2) patients with late-onset T1D, and (3) patients with LADA. Differences in* H. pylori* prevalence were compared using Pearson *χ* ^2^ test or Cochran-Armitage test for a linear trend in proportions. All statistical analyses were carried out using SPSS statistical software (version 16.0, Chicago, IL, USA) and *p* values lower than 0.05 were considered statistically significant.

## 3. Results

From the total of 495 serum samples tested for* H. pylori* infection, there were 156 patients with T2D, 105 with late-onset T1D, and 234 with latent autoimmune diabetes. The overall seropositivity of* H. pylori* infection was 49% in the T2D group, 45% in T1D group, and 52% in LADA group (Table 1). There was no significant difference in the overall seroprevalence of* H. pylori* infection among males and females within each of the three groups. The previously described birth cohort effect in the acquisition of* H. pylori* infection was evident [22].

**Figure 1:**
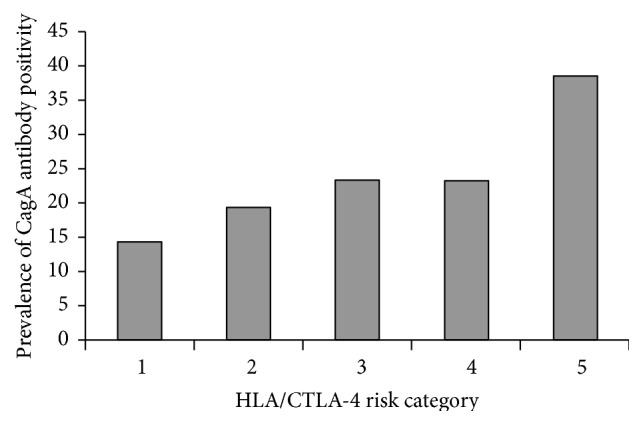
Prevalence of CagA antibody positivity in LADA patients stratified according to a combined HLA/CTLA-4 genetic risk score. No patients were found in the HLA low/CTLA-4 low category. The categories of genetic risks were defined as follows: 0 (HLA low/CTLA-4 low); 1 (HLA low/CTLA-4 high); 2 (HLA intermediate/CTLA-4 low); 3 (HLA intermediate/CTLA-4 high); 4 (HLA high/CTLA-4 low); 5 (HLA high/CTLA-4 high).

Among 245 who tested positive for* H. pylori*, 89 patients (36%) were positive for IgG antibody anti-CagA. Patients with autoimmune diabetes consistently had higher prevalence of CagA positive strains compared with T2D (Table 2). The prevalence of CagA antibodies increased linearly, though not significantly, based on the HLA/CTLA-4 score haplotypes, known to confer a high or intermediate risk of autoimmune diabetes. On the contrary, IgG anti-*H. pylori* did not vary significantly (Figure 1).

## 4. Discussion

The link between* H. pylori* infection and diabetes remains controversial [17, 23–30]. Although an increased susceptibility to* H. pylori* could explain its higher prevalence in patients with diabetes, both diseases could share common susceptibility genes.

In this study, we found that anti-CagA antibodies in LADA patients vary according to the presence of genetic variants previously associated with the risk of autoimmune diabetes. While differences in IgG anti-*H. pylori* prevalence were not significant across the HLA class II or CTLA-4 genotypes, CagA antibody prevalence was found to be higher, though not significantly. However,* H. pylori* virulence factor might confer a high or intermediate risk for autoimmune diabetes in carriers of HLA haplotypes and carriers of the CTLA-4 6230G allele (AG and GG genotypes). These results reflect what has already been observed in the case of anti-GAD65 antibodies whose levels were significantly increased in LADA patients carrying the high-risk HLA-DR3 haplotypes [31]. Similarly, it may be hypothesized that carriers of genetic variants, associated with an increased risk for autoimmune disease, may have an intrinsic predisposition also to develop a stronger immune response against infectious agents, including* H. pylori*, compared to subjects who do not carry the gene variants. This could be consistent with the findings of our study where the prevalence of CagA positivity increased with the number of HLA/CTLA-4-related genetic risks. The capacity of high-risk HLA and CTLA-4 gene variants to influence binding and later presenting autoantigens (including pancreatic beta cells) to autoreactive T lymphocytes [32], as well as the production of antibodies by B cells, may be different depending on the cell repertoire, resulting in distinct antibody response observed for anti-*H. pylori* and anti-CagA. This conjecture deserves to be tested in larger cohorts.

The Sardinian population is characterized by high prevalence of genetic disorders including T1D [33]. Similarly, very high prevalence of* H. pylori* has been demonstrated in healthy children and adults [34]. In a previous study, prevalence of* H. pylori* infection in diabetic patients (insulin-dependent, or type 1, and non-insulin-dependent, or type 2, diabetes mellitus) was compared with a healthy control group [12]. The diagnosis of diabetes was confirmed for at least one year earlier.* H. pylori* status was evaluated by using an ELISA for anti-*H. pylori* immunoglobulin G [12]. We found no significant differences in the prevalence of* H. pylori* infection in any age group; however, the prevalence of* H. pylori* was higher among healthy children than among children with T1D (25% versus 9%) [12]. Our current study also showed no difference in the prevalence of* H. pylori* infection between patients with autoimmune and nonautoimmune diabetes although prevalence of infection with more virulent strains was increased in patients with autoimmune diabetes. Impairment of cellular and humoral immunity in diabetic patients could enhance an individual's susceptibility to acquire* H. pylori* infection [35] and altered glucose metabolism might facilitate* H. pylori* colonization in the gastric mucosa [36]. For example, diabetes-induced reduction of gastrointestinal motility and acid secretion may promote pathogen colonization and infection rate in the gut [9].* H. pylori* infection may also contribute to the development of diabetes as the infection is associated with chronic low-grade inflammation with upregulation of cytokines such as C-reactive protein, tumor necrosis factor, and interleukin 1*β*, which may influence insulin action and pancreatic *β* cell secretion. In the same time,* H. pylori*-induced gastritis affects the secretion of gastric hormones, including leptin, ghrelin, gastrin, and somatostatin, which could affect insulin sensitivity and glucose homeostasis [37–39].* H. pylori* is one of the most infectious agents proposed as an agent triggering an autoimmune response and molecular mimicry is one of the several mechanisms that have been suggested in an attempt to explain the extraintestinal manifestations of* H. pylori* infections [3, 4]. Most* H. pylori* infected individuals produce antibodies to a variety of* H. pylori* antigens. An antibody response may also be seen against autoantigens, including IL-8, antral epithelium, homologous host, and bacterial epitopes (e.g., Lewis X, lipopolysaccharide, and heat shock protein) [40]. For example,* H. pylori* infection has been associated with the pathogenesis of autoimmune thrombocytopenia and autoimmune pancreatitis. A study showed homology with an amino acid sequence of plasminogen-binding protein (PBP) of* H. pylori* and with ubiquitin-protein ligase E3 component n-recognin 2, an enzyme highly expressed in acinar cells of the pancreas. Antibodies against the PBP peptide were detected in 95% of patients with autoimmune pancreatitis and in 10% of patients with pancreatic cancer [41].

CagA positive strains cause more intense tissue inflammation and cytokine production and are specifically related to the pathogenesis of autoimmune thrombocytopenia. More specifically, bacteria that express CagA are potent inducers of IL-8 [34]. CagA positivity is also associated with more severe clinical outcomes such as duodenal ulcers, precancerous lesions, and gastric cancer [42]. The increased prevalence of IgG anti-CagA in autoimmune diabetes might be related to possible cross-reactivity of IgG anti-CagA with host's beta cell antigens. In fact, immunoreactivity of anti-CagA antibodies was demonstrated with many human antigens, such as vessel, smooth muscle cells, and fibroblasts-like cells in intimal atherosclerotic plaques [43, 44], and therefore it could also occur in the case of beta cell antigens. If this hypothesis is confirmed, it is tempting to speculate that patients who suffer from an infection by virulent CagA positive* H. pylori* strains may elicit an immune-mediated response against specific self-antigens, including those associated with pancreatic beta cells, thereby displaying increased susceptibility to autoimmune diabetes.

## 5. Conclusions

Although the cross-sectional design of the study does not permit discerning a cause-effect relationship between* H. pylori* infection and diabetes, higher prevalence of virulent strains was observed among patients with autoimmune diabetes. Prevention and progression of diabetes could be an additional long term benefit of* H. pylori* eradication.

## Figures and Tables

**Table 1:** Unadjusted odds ratio and 95% confidence interval for frequency of *H. pylori* antibodies in the adult population according to the three study groups: late-onset type 1 diabetes, latent autoimmune diabetes, and type 2 diabetes, stratified by birth cohorts.

Variable	Type 1 Number (Hp%)^§^	OR^@^ (95% CI^†^)	LADA Number (Hp%)^§^	OR^@^ (95% CI^†^)	Type 2 Number (Hp%)
Birth cohort (y)					
	1 (100)	—	14 (43)	—	0
1930–1939	19 (68)	3.25 (0.43–24.84)	52 (53)	1.62 (0.25–10.51)	5 (40)
1940–1949	24 (50)	0.68 (0.27–1.69)	86 (58)	0.86 (0.47–1.58)	84 (54)
1950–1959	24 (42)	0.63 (0.24–1.69)	50 (50)	0.82 (0.37–1.80)	48 (50)
1960–1969	23 (39)	1.93 (0.47–7.88)	30 (47)	2.63 (0.69–10.02)	15 (27)
	14 (14)	—	6 (33)	—	4 (0)
Total	105 (45)	0.75 (0.46–1.23)	234 (52)	0.96 (0.64–1.43)	156 (49)

Gender					
Male	55 (44)	0.74 (0.37–1.46)	108 (52)	1.01 (0.57–1.78)	85 (54)
Female	50 (46)	0.95 (0.46–1.96)	126 (52)	1.14 (0.64–2.02)	71 (46)

^§^Percentage positive for *Helicobacter pylori *infection.

^**@**^Odds ratio; ^†^confidence interval.

**Table 2:** Distribution of CagA status among the three study groups.

	Autoimmune diabetes		Nonautoimmune diabetes
	Type 1	LADA	Type 2
	(*n* = 105)	(*n* = 234)	(*n* = 156)
Hp negative	58 (55%)	112 (48%)	80 (51%)
Hp positive, CagA negative	26 (25%)	73 (31%)	57 (37%)
Hp positive, CagA positive	21 (20%)	49 (21%)	19 (12%)
CagA prevalence in Hp positive patients	45%	40%	25%

Hp positive, autoimmune versus nonautoimmune diabetes: *χ* ^2^ = 0.0055; *p* = 0.815.

CagA positive, autoimmune versus nonautoimmune diabetes: *χ* ^2^ = 1.556; *p* = 0.212.

CagA positive/Hp positive, type 1 versus type 2 diabetes: *χ* ^2^ = 5.125; *p* = 0.024.

CagA positive/Hp positive, LADA versus type 2 diabetes: *χ* ^2^ = 4.775; *p* = 0.028.
